# Parasites of firebugs in Austria with focus on the “micro”-diversity of the cosmopolitan trypanosomatid *Leptomonas pyrrhocoris*

**DOI:** 10.1007/s00436-023-08080-2

**Published:** 2023-12-11

**Authors:** Alexei Yu. Kostygov, Ľubomíra Chmelová, Julia Reichl, Alexandra Jászayová, Jan Votýpka, Hans-Peter Fuehrer, Vyacheslav Yurchenko

**Affiliations:** 1 Faculty of Science, University of Ostrava, Ostrava, Czechia; 2 Institute of Parasitology, University of Veterinary Medicine Vienna, Vienna, Austria; 3 Institute for Medical Microbiology and Hygiene, AGES - Austrian Agency for Health and Food Safety, Vienna, Austria; 4 Faculty of Science, Charles University, Prague, Czechia; 5 Institute of Parasitology, Biology Centre, Czech Academy of Sciences, České Budějovice, Czechia

**Keywords:** Firebugs, *Pyrrhocoris apterus*, Mermithidae, *Blastocrithidia*

## Abstract

In this work, we investigated parasites of the firebug *Pyrrhocoris apterus* in Austria and demonstrated that in addition to the extensively studied *Leptomonas pyrrhocoris*, it can also be infected by *Blastocrithidia* sp. and by a mermithid, which for the first time has been characterized using molecular methods. This diversity can be explained by the gregarious lifestyle, as well as the coprophagous and cannibalistic behavior of the insect hosts that makes them susceptible to various parasites. In addition, we showed no tight association of the *L*. *pyrrhocoris* haplotypes and geographical locations (at least, considering the relatively small scale of locations in Austria) implying that the natural populations of *L*. *pyrrhocoris* are mixed due to the mobility of their firebug hosts.

**Supplementary Information:**

The online version contains supplementary material available at 10.1007/s00436-023-08080-2.

## Introduction

In the last decades, the use of molecular methods has revealed an enormous and unsuspected genetic diversity of protists, including parasitic members of this group (Burki et al. 2021). Although most of analyses were based on the conservative SSU rRNA gene sequences, they repeatedly demonstrated large genetic heterogeneity within various morphospecies. However, it remains to be established whether this is accompanied by functional diversity or represents just an outcome of the neutral mutations (Caron and Hu 2019; Foissner 2007).

The term “microdiversity” is predominantly used for prokaryotes and refers to the diversity of phylogenetically related, but physiologically distinct, groups of microbes (Larkin and Martiny 2017). Traditionally, species were considered as units of diversity; however, in the case of microbes, the formal definition of a species requires its isolation in pure culture, phenotypic characterization, and (nowadays) genome sequencing (Browne et al. 2016; Leon et al. 2014). Microdiversity and the existence of various ecotypes of microorganisms have been proposed to confer stability to microbial ecosystems but could also explain the coexistence of different genetic lineages of symbiotic organisms in the host, from mutualistic and commensal to parasitic (Larkin and Martiny 2017).

In biology, model organisms are carefully selected and studied to provide insights into fundamental processes and general principles applicable to a wide range of species (Muller and Grossniklaus 2010). They should have short generation times, be easy to manipulate genetically, and have sequenced and well-annotated genomes. Our long-term efforts have been focused on the establishment of a nonpathogenic model monoxenous (with one host) trypanosomatid, as a supplement to human-pathogenic dixenous (shuttling between two hosts) kinetoplastids of the genera *Trypanosoma* and *Leishmania* (Butenko et al. 2018; Flegontov et al. 2016; Macedo et al. 2023; Votýpka et al. 2012b).

The parasitic flagellate *Leptomonas pyrrhocoris* belongs to the subfamily Leishmaniinae, which, in addition to other *Leptomonas* spp., encompasses five monoxenous and three dixenous genera, including *Leishmania* (Kostygov and Yurchenko 2017). This species was originally isolated from the midgut of a firebug, *Pyrrhocoris apterus*, but the range of suitable hosts also includes other members of the family Pyrrhocoridae from the genera *Pyrrhocoris* and *Scantius* in Europe and the Mediterranean, as well as *Dysdercus* in Asia, Africa, and the Neotropics (Maslov et al. 2007; Votýpka et al. 2012b; Votýpka et al. 2020; Votýpka et al. 2010). It has been proposed that *L*. *pyrrhocoris* originated in the latter region (Votýpka et al. 2012b). Of all known monoxenous trypanosomatids, this species represents the most attractive model. In general, its pyrrhocorid hosts are virtually omnipresent, which allows to find this species in different corners of the world. However, what is more relevant to us is that in Europe, it inhabits *Pyrrhocoris* apterus, a unique insect from several points of view, making it a very popular biological model *per se*. Indeed, this is a highly abundant and easily recognizable species with gregarious lifestyle, virtually no natural predators, and the feeding strategies combining phytophagy with cannibalism, scavenging, and coprophagy (Socha 1993). These peculiarities of the firebugs determine high prevalence of *L*. *pyrrhocoris* in the host populations and, at the same time, its separation from the majority of other trypanosomatids circulating in nature (Frolov et al. 2021).

It has been demonstrated that *L*. *pyrrhocoris* shows a genetic variation that correlates with its geographical distribution suggesting independent evolution in different regions (vicariance) instead of global homogenization through multiple migrations (Votýpka et al. 2012b). However, an opposite model has been posited recently as the results of a viral screening in isolates of *L*. *pyrrhocoris* from the continental Europe. It documented no geographic pattern in the diversity of *Leppyr*TLV1, the highly prevalent virus specific to this trypanosomatid (Macedo et al. 2023), indirectly implying that, at least in the Central Europe, genetic variation of *L*. *pyrrhocoris* does not correlate with its geographical distribution. Therefore, in the current study, we decided to switch from the global diversity of *L*. *pyrrhocoris* to the local one and focused on a geographically limited area, specifically a single European country—Austria. Using the highly variable molecular marker, Spliced Leader RNA (SL RNA) gene, we revealed microdiversity of the flagellates even at the level of individual firebugs and demonstrated that distribution of haplotypes does not have any geographic pattern. In addition, we detected other *P*. *apterus* parasites (a trypanosomatid *Blastocrithidia* sp. and a mermithid *Amphimermis* sp.) not previously recorded in these bugs.

## Material and methods

### Material collection

**Fig. 1:**
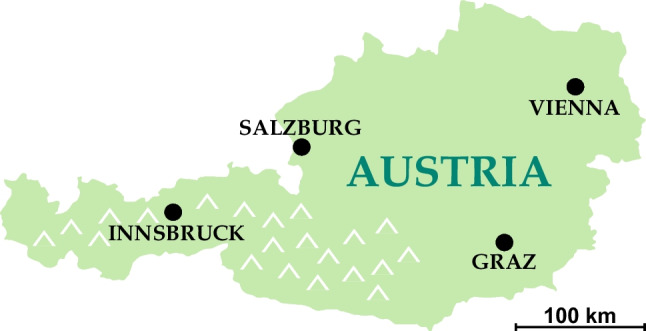
Map of Austria showing the cities, where the firebugs were collected

Firebugs (*Pyrrhocoris apterus*) were collected by handpicking in four Austrian cities: Vienna, Graz, Salzburg, and Innsbruck (Fig. 1). Two to four collection sites were chosen in each city (Table 1). The insects were maintained in individual perforated Eppendorf tubes and dissected within 1 to 2 days after capture with microscopic inspection of gut contents and preservation of infected material as described earlier (Kostygov et al. 2022). In the case of mermithid, the entire worm was preserved for DNA isolation separately from the remaining gut contents.

**Table 1:** Summary of collected material

Date	City	Location	Coordinates	Prevalence
July 24, 2022	Vienna	(1) Single tree near Rossauer barracks	48°13′2″N, 16°22′3″E	0/12 (0%)
		(2) Small garden near Augarten bridge	48°13′7″N, 16°22′18″E	6/8 (75%)
		(3) Augarten, entrance from Untere Augartenstrasse	48°13′19″N, 16°22′37″E	7/7 (100%)
		(4) Kaiserwiese	48°12′59″N, 16°23′38″E	2/2 (100%)
July 27, 2022	Graz	(1) Augarten, tree at the very entrance	47°3′50″N, 15°26′6″E	1/5 (20%)
		(2) Stadtpark, memorial stone	47°4′32″N, 15°26′39″E	12/24 (50%)
July 29, 2022	Salzburg	(1) Moenchsberg, single tree near Siegmundstor West	47°47′55″N, 13°2′19″E	14/16 (88%)
		(2) Moenchsberg, lawn surrounded with trees	47°47′41″N, 13°2′29″E	5/6 (83%)
		(3) Moenchsberg, tree on the alley	47°47′47″N, 13°2′23″E	5/9 (55%)
July 30, 2022	Innsbruck	(1) Gramarstrasse	47°17′10″N, 11°23′49″E	24/29 (83%)
		(2) Alpenzoo, near wolfs	47°16′54″N, 11°23′53″E	4/4 (100%)
		(3) Alpenzoo, near the cross between moose and ibex	47°16′55″N, 11°23′50″E	5/5 (100%)
			Total	84/126 (67%)

### DNA isolation, PCR, and sequencing

Total genomic DNA from collected parasite-containing samples was isolated using GeneJET Genomic DNA Purification Kit (Thermo Fisher Scientific, Waltham, USA) following the protocol of the manufacturer. All DNAs from trypanosomatid-containing samples were subjected to the amplification of partial (~830 bp) 18S rRNA gene using the primers 1127F and 1958R (Ganyukova et al. 2020). The obtained PCR fragments were sequenced directly with the same primers. Out of total 84 infected specimens, 37 samples representing different locations were selected for further analysis of SL RNA using the primers M167 and M168 (Westenberger et al. 2004) and PCRBIO HiFi Polymerase (PCR Biosystems, London, UK). The obtained PCR products were excised from an agarose gel, purified with GeneJET Gel Extraction Kit and cloned using CloneJET PCR Cloning Kit (both from Thermo Fisher Scientific). One to seven clones were sequenced per sample. The GenBank accession numbers of SL RNA gene sequences are OR619935-OR620081.

The two DNA samples obtained from mermithids were used for amplification of the cytochrome oxidase subunit I (*COI*) gene using the “universal” invertebrate primers L1490 and H2198 (Folmer et al. 1994) along with the 18S rRNA gene. To specifically amplify the latter gene from mermithids, universal eukaryotic primers A and B (Medlin et al. 1988) were combined with the newly designed primers MeriR1 (5′–GCTATCAATCTGTCAATCCTTATTG–3′) and MeriF1 (5′–TAGAGGTGAAATTCTTGGATCGCA–3′), respectively, which produced overlapping fragments. Both gene fragments were sequenced directly using amplification primers. The obtained sequences were deposited to the GenBank under the accession numbers OR614373-OR614374 (18S rRNA gene) and OR612938-OR612939 (*COI* gene).

### Phylogenetic analyses

The trypanosomatid 18S rRNA gene sequences obtained in this work were compared to each other and to those in GenBank using BLASTn search algorithm. For the identification of SL RNA gene haplotypes, DnaSP v.6 software was used (Rozas et al. 2017). The haplotype median-joining network was inferred in PopART v. 1.7 (Leigh et al. 2015). Considering the high probability that the poly(T) region (ranking from 16 to 36 Ts) contains reading errors due to polymerase slippage, this region was not included in the haplotype classification and phylogenetic analysis. For the inference of the phylogenetic tree, all the obtained SL RNA gene sequences were combined with those available for *L*. *pyrrhocoris* in GenBank and aligned by MUSCLE v. 3.8.31 (Edgar 2004) with default parameters, producing a matrix of 1064 characters and 198 sequences. Phylogenetic inference was performed using RAxML 8.2.1 (Stamatakis 2014) with 1000 bootstrap replicates and other parameters left in their default states.

The *COI* sequences obtained in this work were submitted for the search in the Barcode of Life Data System (https://boldsystems.org/) using the “Species Level Barcode Records” and “All Barcode Records” databases. The 18S rRNA gene sequences of mermithids were used for BLASTn search against the nr database of GenBank, and the sequences of their 17 closest relatives were retrieved. All sequences were aligned using the E-INS-i algorithm in MAFFT v. 7.490 (Katoh and Standley 2013). The resulting alignment was used for the maximum likelihood tree inference in IQ-TREE v. 2.2.0 (Minh et al. 2020) under the TN + F + I + G4 substitution model, as automatically selected by the built-in ModelFinder module and branch support estimated with 1000 ultrafast bootstrap replicates.

## Results and discussion

Of 126 adult firebugs, 85 (67%) were positive for trypanosomatids based on their dissection and microscopic examination, which was subsequently confirmed by PCR and sequencing. While the total prevalence in males and females was almost identical (68% vs 67%, respectively), some differences were detected between individual micro-populations (Table 1). In most cases, the infection rates were high (50–100%); however, there were two exceptions. In one of the localities (a lonely tree in the street in Vienna), all 12 dissected firebugs were trypanosomatid-free, which could be due to the founding of this small micropopulation by a single female. In the whole Augarten park in Graz, we were able find only five firebugs, all under a single tree, of which only one proved to be infected. Apparently, this resulted from the paucity of the bugs in this place.

We obtained partial sequences of 18S rRNA from all positive specimens. However, all these sequences were identical and shared 100% similarity to the already published sequences of *L*. *pyrrhocoris*, which was in line with the previous data on the high prevalence of this parasite in firebugs (Votýpka et al. 2012b). However, the analysis of 146 SL RNA gene sequences revealed that five of them (corresponding to two samples from Graz and Salzburg) belonged to the undescribed species *Blastocrithidia* sp. TU17 recorded predominantly in meadow bugs (Miridae) (Kozminsky et al. 2015; Votýpka et al. 2012a; Westenberger et al. 2004). The absence of the 18S rRNA signal for *Blastocrithidia* sp. in these samples (no sign of admixture in the chromatograms) suggests that the number of the cells of this parasite there was very low. Therefore, most likely, these two cases correspond to non-specific presence of this *Blastocrithidia* sp. in firebugs, probably even not as vegetative stages but the resting cyst-like amastigotes, which are inherent to *Blastocrithidia* spp. (Kostygov et al. 2021). These cells are able to survive in adverse environmental conditions for several years (Frolov et al. 2021) and could be accidentally ingested by the firebugs. Surprisingly, the second most frequent and highly specific parasite of *Pyrrhocoris apterus*, *Blastocrithidia papi* (Frolov et al. 2017; Frolov et al. 2018), was not detected in any studied sample.

**Fig. 2:**
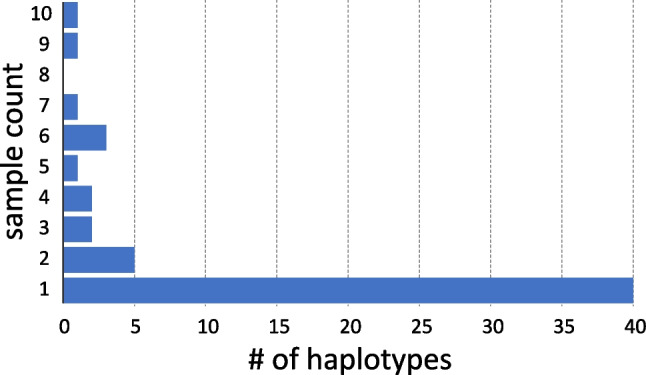
SL RNA gene haplotype frequency in studied isolates of *L*. *pyrrhocoris*

**Fig. 3:**
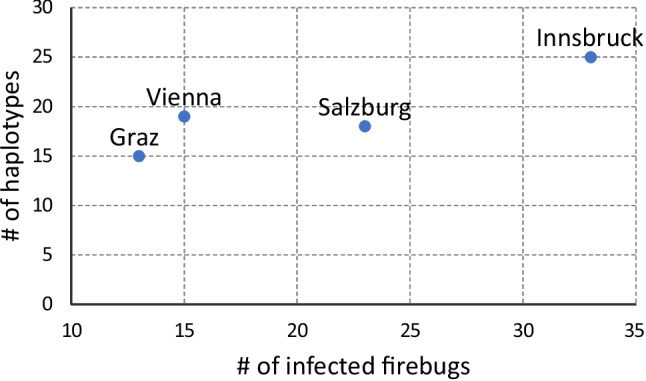
Correlation of haplotype number with the number of infected firebugs

**Fig. 4:**
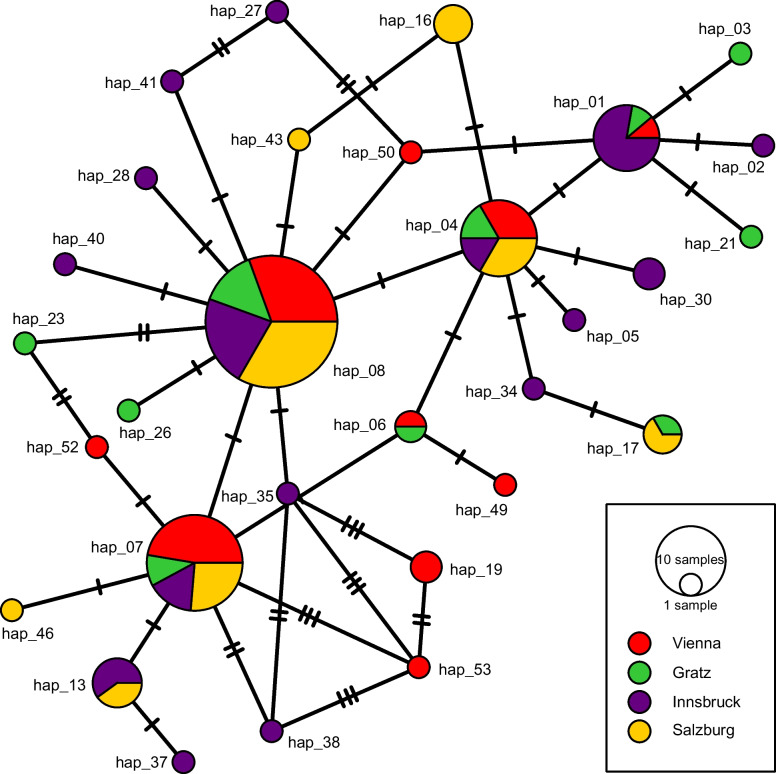
A median-joining haplotype network of SL RNA gene sequences of Austrian isolates of *Leptomonas pyrrhocoris*. Circles represent individual haplotypes; their color indicates the city of origin, while their size is proportional to the haplotype frequency. Number of substitutions is depicted by crossing marks

The remaining 141 SL RNA gene sequences belonged to *L. pyrrhocoris* and constituted 56 haplotypes. Of them, 40 haplotypes were unique within the dataset, and the maximum number of firebugs sharing the same haplotypes (hap_10) was ten (Fig. 2). Conversely, we documented up to five different haplotypes within a single individual. As expected, the number of detected haplotypes increased with the number of infected individuals at the respective sites and ranged from 15 (in Graz) to 25 (Innsbruck) (Fig. 3). The inferred haplotype network showed no clear pattern with respect to the geographical origin of infected firebugs (Fig. 4). In addition to regular random substitutions, about 17% of the sequences (24/141) featured a 196 bp deletion in the intergenic region making such SL repeat significantly shorter than others (~870 vs 1070 bp). Thus, our results suggest that there is a considerable genetic variation of *L*. *pyrrhocoris* at this small geographic scale, and the absence of physical barriers allows the intermixing of firebug populations and their parasites leading to the diversity even within a single individual. We speculate that individual lineages (here, proxied by haplotypes) phenotypically differ that allows them to make suitable use of either hosts with different physiological status or different parts of the microenvironment within a single host. We posit that the natural populations of *L*. *pyrrhocoris* are mixed due to the mobility of the firebug hosts. Although in the previous study (Macedo et al. 2023), it was only possible to use indirect arguments (occurrence of various viruses in different parasite strains); here, we can demonstrate it directly on the *L*. *pyrrhocoris* haplotypes.

**Fig. 5:**
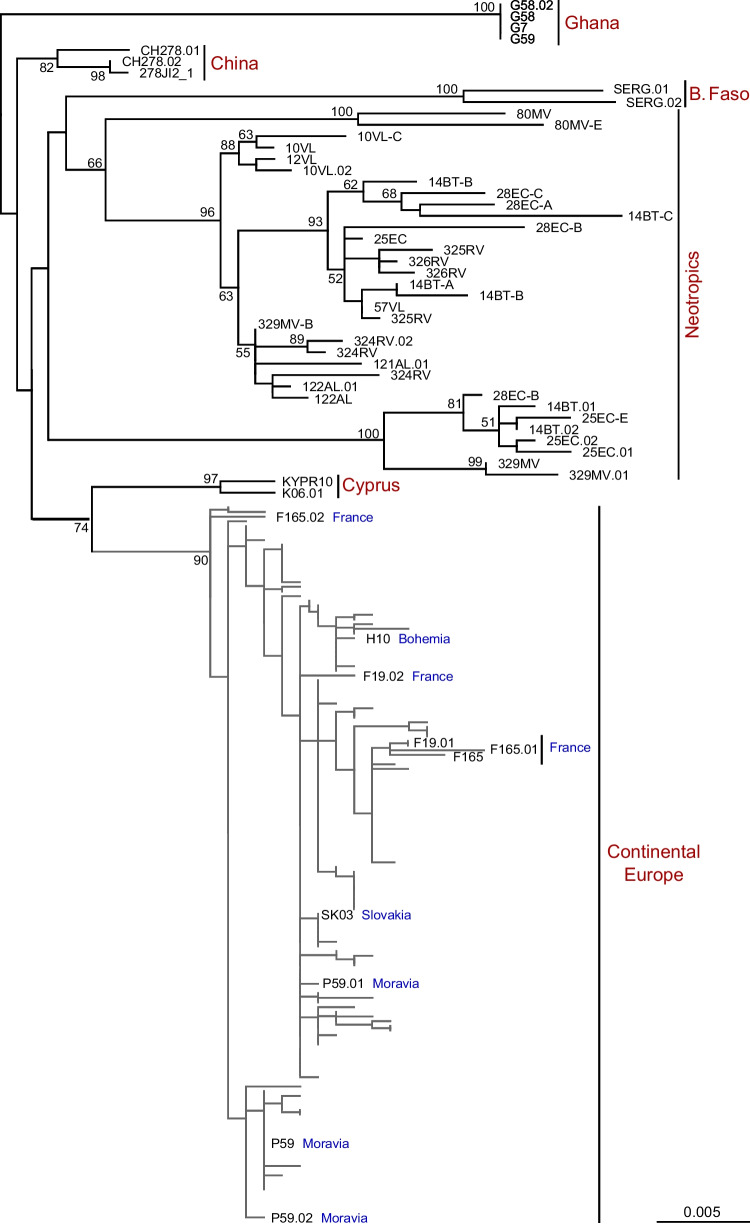
Maximum likelihood phylogenetic tree of *Leptomonas pyrrhocoris* SL RNA gene sequences. Numbers at branches correspond to bootstrap supports; values below 50% are not shown. The vertical distances between the taxa in the subtree of Continental Europe (in gray) are decreased. All unlabeled branches correspond to the isolates from Austria. Scale bar shows number of substitutions per site

The newly obtained SL sequences from the Austrian firebugs were also analyzed along with the sequences already available in the GenBank database. As in our previous study (Votýpka et al. 2012b), the macro-biogeographic pattern of the intraspecies variability of the *L*. *pyrrhocoris* lineages was clear (Fig. 5). These findings further support the hypothesis that while geographical differences can be observed on a global scale, a large number of haplotypes coexist locally due to the (active or passive) mobility of their hosts (Macedo et al. 2023).

We cannot rule out a possibility that the observed diversity of SL sequences co-occurring even in one host specimen could be explained not only by a mixture of different strains/haplotypes but also by intragenomic variation. However, a comparison of all 30 SL RNA gene repeats in the chromosome-level assembly of the H10 isolate of this species (scaffold LGTL01000027, assembly ASM129339v1) did not reveal any sequence variation between them (except the truncation of terminal copies). As for the effect of PCR errors, we attempted to minimize their rate by using a proofreading polymerase with the accuracy being 50× higher than that of the regular Taq polymerase.

**Fig. 6:**
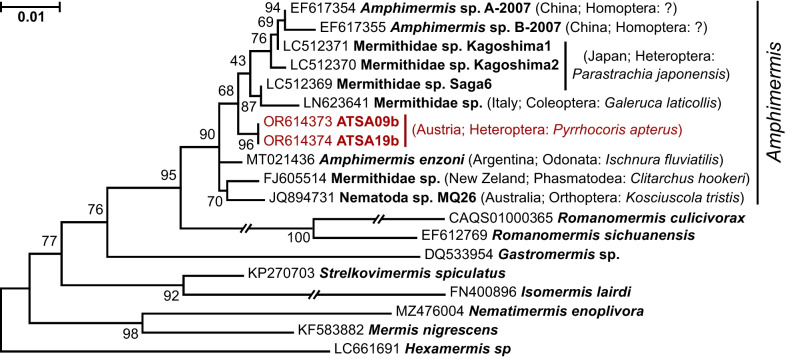
Maximum likelihood phylogenetic tree showing position of the mermithids documented in this study. The two isolates from firebugs are shown in crimson color. The tree was rooted according to the topology of a previously inferred tree (Kakui and Shimada 2022). The numbers at branches are ultrafast bootstrap supports. Double crossed branches are at 50% of their length. Scale bar corresponds to the number of substitutions per site

In addition to trypanosomatid species, two firebugs were infected with unidentified nematode species of the family Mermithidae (both were from the same micropopulation in Salzburg). The 18S rRNA gene sequences from both samples were identical; BLAST search identified the closest sequence (98.6%) in GenBank originated from a mermithid found in an Australian grasshopper *Kosciuscola tristis* (Umbers et al. 2014). The phylogenetic inference based on this gene demonstrated that the worms from firebugs belonged to a clade, some members of which have been previously identified as representatives of the genus *Amphimermis* (Fig. 6). Surprisingly, some of the related mermithids have been previously found in *Parastrachia japonensis*, a shield bug from Japan, which convergently acquired the same coloring pattern and gregarious habit (Iryu et al. 2020). Although both 18S rRNA gene sequences of mermithids from firebugs were identical, those for COI slightly differed (99.6% identity), suggesting acquisition from different sources. The search in the “Species Level Barcode Records” database did not reveal any significant similarity allowing the identification only up to family. However, the extended database “All Barcode Records” included sequences of the same species (as can be judged by 99.2–99.4% similarity), which originated from Germany and Bulgaria (Fig. S1). Only one of these sequences was accompanied by a photo in the database, depicting a brachyceran fly, suggesting a potential source of the infection in firebugs. The records of mermithids in true bugs (Heteroptera) are rare. In addition to what has been already mentioned above, the occurrence of mermithids has been relatively recently documented in the members of the family Pentatomidae in Japan (Watanabe et al. 2020) and USA (Fuxa et al. 2000; Stubbins et al. 2015; Stubbins et al. 2016), as well as in Reduviidae in Brazil (Martins et al. 2020). Among hundreds of dissections of firebugs that we have conducted in the past decades, adult mermithids have never been detected. However, such records exist in the old literature (Southwood and Leston 1959; van Zwaluwenburg 1928).

## Conclusions

Here, we demonstrated that *P*. *apterus* harbors numerous parasites of several types. In addition to the extensively studied *L*. *pyrrhocoris*, we have also identified infection by *Blastocrithidia* sp. and by a mermithid, which for the first time has been characterized using molecular methods. This can be explained by the gregarious lifestyle, as well as the coprophagous and cannibalistic behavior of the insect hosts that makes them susceptible to various parasites.

Another important conclusion of our work is that no tight association of the *L*. *pyrrhocoris* haplotypes and geographical locations (at least, considering the relatively small scale of locations in Austria) was detected. This means that the natural populations of *L*. *pyrrhocoris* are mixed due to the mobility of their firebug hosts.

### Supplementary information

ESM 1 (PDF 39 kb)

## Data Availability

All sequence data obtained in this work were submitted to GenBank with the following accession numbers: OR614373-OR614374 (18S rRNA gene of mermithids), OR612938-OR612939 (*COI* gene of mermithids), OR619935-OR620081 (SL RNA gene sequences of *L*. *pyrrhocoris*).
