# Supplementary figures and images for: Parasites of firebugs in Austria with focus on the “micro”-diversity of the cosmopolitan trypanosomatid Leptomonas pyrrhocoris
Source: Parasitol Res. 2023 Dec 11;123(1):27. doi: 10.1007/s00436-023-08080-2 (PMC10710968; doi:10.1007/s00436-023-08080-2)

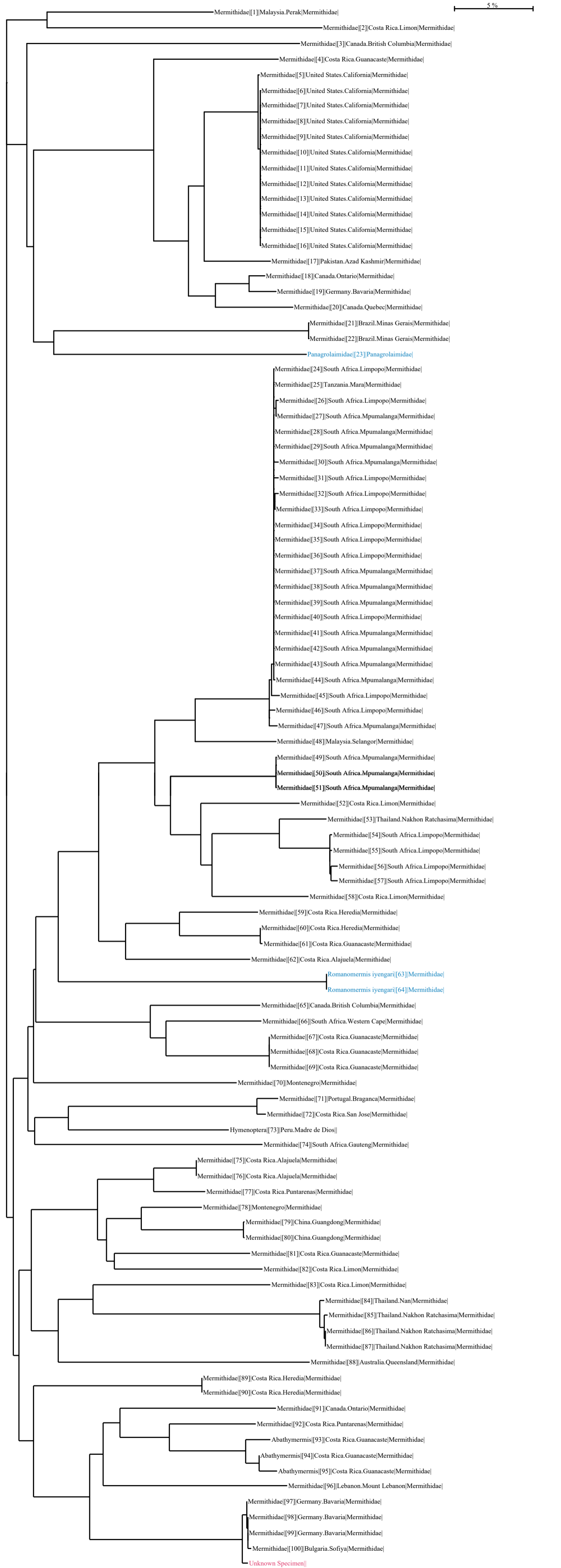

Supplement: Supplementary file 1 — (PDF 39 kb) [file 436_2023_8080_MOESM1_ESM.pdf]
